# Analysing the contribution of trees and green spaces to household nutrition security in eThekwini, KwaZulu-Natal, South Africa

**DOI:** 10.3389/fsufs.2024.1451656

**Published:** 2024-11-25

**Authors:** Qhelile Ntombikayise Bhebhe, Muthulisi Siwela, Temitope O. Ojo, Simphiwe Innocentia Hlatshwayo, Tafadzwanashe Mabhaudhi, Rob Slotow, Mjabuliseni S. C. Ngidi

**Affiliations:** 1 African Centre for Food Security, College of Agriculture, Engineering and Science, School of Agricultural, Earth and Environmental Sciences, University of KwaZulu-Natal, Pietermaritzburg, South Africa; 2 Centre for Transformative Agricultural and Food Systems, College of Agriculture, Engineering and Science, School of Agricultural, Earth and Environmental Sciences, University of KwaZulu-Natal, Pietermaritzburg, South Africa; 3 Dietetics and Human Nutrition, School of Agricultural, Earth and Environmental Sciences, University of KwaZulu-Natal, Pietermaritzburg, South Africa; 4 Department of Agricultural Economics, Obafemi Awolowo University, Ile, Nigeria; 5 Disaster Management Training and Education Centre for Africa, University of the Free State, Bloemfontein, South Africa; 6 Centre on Climate Change and Planetary Health, London School of Hygiene and Tropical Medicine, London, United Kingdom; 7 Centre for Functional Biodiversity, School of Life Sciences, College of Agriculture, Engineering and Science, University of KwaZulu-Natal, Pietermaritzburg, South Africa; 8 Department of Agricultural Extension and Rural Resource Management, College of Agriculture, Engineering and Science, School of Agricultural, Earth and Environmental Sciences, University of KwaZulu-Natal, Pietermaritzburg, South Africa

**Keywords:** diets, nutrition, Food Consumption Score, trees and green spaces, food groups, household nutrition security

## Abstract

Food from trees and green spaces can diversify diets and enhance food and nutrition security for households. However, sourcing food from these areas often receives little attention in addressing nutrition issues. This study assessed the contribution of trees and green spaces to household food consumption and nutrition in the eThekwini Municipal Area (EMA) located in KwaZulu-Natal South Africa, focusing on the Osindisweni and Maphephetheni communities, which are biologically diverse and face high poverty, unemployment, and food insecurity. Using stratified random sampling, 280 households were selected to complete questionnaires. Additionally, two Focus Group Discussions (FDG’s) and key informant interviews were conducted with community members and municipal representatives. Data were analyzed using descriptive statistics, the Household Food Consumption Score (FCS), Ordered Logistic Regression and a thematic analysis was done to analyse responses from Focus Group Discussions. The results showed that 93.6% of households consumed acceptable diets, with only 5.0% in the borderline and 1.4% in the poor categories. Specifically, Osindisweni and Maphephetheni households reported 93.3% and 93.7% acceptable diets, respectively. Ordered logistic regression indicated that both cultivated and uncultivated green spaces, household size, number of dependants, as well as access to training, agricultural assistance, extension, and advisory services negatively correlated with nutrition security. While communities recognized the contributions of trees and green spaces, they believed that these sources alone were insufficient. It is concluded that consumption of products from trees and green spaces likely did not improve the nutrition security of the households. To improve household nutrition security in eThekwini, it is vital to foster collaboration among stakeholders, including nutritionists and extension agents. Strengthening the knowledge of extension officers regarding the harvesting and consumption of food from trees and green spaces is crucial for disseminating effective guidance to households, thereby enhancing nutrition outcomes.

## 1 Introduction

The prevalence of malnutrition and hunger remains high, particularly in developing nations, despite food and nutrition security improvements over the past few decades (Masuku et al., 2017). Nearly 2 billion individuals worldwide are undernourished, primarily due to the inadequate intake of micronutrients such as vitamins, iron, and zinc (Boatemma et al., 2018). Most recent reports (Boatemma et al., 2018; Fanzo et al., 2017; Hlpe, 2017; Dlamini, 2020) show an increase in food and nutrition insecurity by 15%, where over 2.3 billion people were food insecure as of 2021 (Mkhize and Sibanda, 2022). Despite malnutrition reductions, 150.8 million children under the age of five are stunted, and another 50.5 million are wasted. Furthermore, the rapidly rising trend in childhood and adult overweight and obesity has emerged as one of the most severe global public health issues of the twenty-first century (Finucane et al., 2011; Collaborators, 2017; Bleich et al., 2018). Sub-Saharan Africa (SSA) has one of the highest rates of child malnutrition in the world [child malnutrition is defined as a pathological state caused by insufficient nutrition, including undernutrition caused by inadequate intake of dietary energy and other vital nutrients, resulting in stunting (low height for age) or wasting (low weight for length) (Bain et al., 2013), and overweight and obesity caused by excessive consumption of dietary energy and reduced levels of physical activity] (Rossouw et al., 2012).

South Africa, a middle-income country with high levels of wealth/economic inequality, is undergoing rapid socio-economic and lifestyle changes that have precipitated a nutritional transition, as well as a high prevalence of overweight/obesity in children (Keino et al., 2014). The dual burdens of undernutrition and overweight/obesity are not evenly distributed across the country, and the health risks associated with malnutrition vary by age, gender, ethnicity, and geographic location (Osgood-Zimmerman et al., 2018). This chronic nutrition insecurity has led to illness and death (Jain et al., 2020). Due to the persistence of food insecurity despite global eradication efforts, researchers have described it as a research problem (Chamberlain et al., 2020; Ofoegbu, 2014). South Africa’s national level of food security is considered “acceptable”, however, a significant number of households and individuals, especially in rural areas, remain food insecure (Boatemma et al., 2018; Sambo et al., 2022). Data at the population level show high levels of nutrition insecurity, from extreme undernutrition to obesity and diet-related diseases (Jain et al., 2020). More so, over half of the adults in South Africa are reportedly overweight, with a higher prevalence of obesity among women than men (Mkhize and Sibanda, 2022; Sambo et al., 2022). The Province of KwaZulu-Natal (KZN), which includes the eThekwini Municipality, is estimated to have a population of ~11.1 million people, most of whom live in rural areas (Stats, 2017). The province has the third-highest incidence of poverty in South Africa, where 47% of the rural population live in extreme poverty, and this appears to be deepening (Munien et al., 2015). To maintain a healthy and nutritious diet, people should consume diversified foods to ensure intake of essential nutrients (Labadarios et al., 2011).

Conversations about improved livelihoods and diets have been centered mainly on agricultural production, improving crop yields, genetic engineering and finding ways to disseminate new technologies and information that will enhance agricultural output. Researchers and policymakers have made the production of energy-rich staple crops such as cereals the main focus in the quest for food and nutrition security (Chamberlain et al., 2020; Turner-Skoff and Cavender, 2019). These staple foods derived from cereals and root tubers contain a limited amount of micro-nutrients and are insufficient in addressing nutritional deficiencies (Govender et al., 2017; Pradhan et al., 2018). Other green spaces such as forests, bushlands and grasslands are rarely featured in such discussions, except when perceived as a space for further agricultural expansion or a resource that needs to be restored and protected because of the consequences of agricultural development (Hlpe, 2017). Following this, there is a need to foster a transition to a more integrated food system that includes natural resource harvesting, such as from trees and green spaces. While steps are being taken to improve this situation, more can still be done. Recognizing their current and potential contribution is necessary to realize and fully account for the potential of all trees and green spaces to sustain human nutrition.

According to Newton et al. (2020), 1.6 billion people lived within 5 kilometers of green spaces in 2012, with two-thirds of them living in low- or middle-income countries and they include indigenous peoples, rural communities, smallholder farmers, and employees of tree enterprises. Despite the fact that, not all have forest access rights, their diets benefit from them in various ways. Nutrient-dense foods from forests and trees contribute significantly to dietary quality and diversity, and thus to human health (Rasolofoson et al., 2018; Rasmussen et al., 2020; Baudron et al., 2019). These are typically harvested in the form of fruits, nuts, edible leaves, and roots; and are rich in protein, iron, calcium, folate, vitamin A, and vitamin C, all of which are often deficient most household diets of poor and vulnerable communities (Kehlenbeck and Jamnadass, 2014). Hence, the complex challenge of nutrition deficiencies underscores the importance of understanding how trees and green spaces can reduce the risks of food and nutrition insecurity.

In this study, trees and green spaces are any open and vegetated green areas that have the potential to contribute to the overall quality and sustainability of household diets (Sardeshpande and Shackleton, 2020). These spaces include forests, parks, grasslands, croplands, wetlands, savannahs, and other terrestrial spaces covered with vegetation and trees (Ellison et al., 2017). Trees and green spaces provide many products and services consumed by people or used to serve their livelihood needs. A plethora of both cultivated and uncultivated plants, fungi and animals are harvested to provide food and medicine, among other necessary items (Chamberlain et al., 2020). Food from trees and green spaces provides calories as well as macro and micro-nutrients essential for human nourishment (Pradhan et al., 2018). More so, they contribute to diversifying the diets of a growing population with increasing food needs (Gitz et al., 2021). Although accounts of the role of trees and green spaces in human nutrition via the direct and indirect provision of food have been illustrated in literature, trees and green spaces are still neglected in formulating solutions meant to deal with food and nutrition insecurity (Jabbar et al., 2021).

There are still limited studies that have focused primarily on the contribution of trees and green spaces to nutrition and food systems despite the agenda set to **“**End poverty in all its forms everywhere” (SDG 1) and “Zero hunger” (SDG 2) (Sivadas, 2022). Hence, this study aims to fill this gap by assessing household nutrition status in the Osindisweni and Maphephetheni communities of eThekwini Municipality and the contribution of trees and green spaces to household nutrition. The specific objective of this study is to determine the contribution of trees and green spaces to household nutrition security.This study hypotheses that trees and green spaces enhance household nutrition security and diets.

## 2 Methods and materials

### 2.1 Description of the study area

The eThekwini Municipal Area (EMA) (Kushitor et al., 2022) is located in the Province of KwaZulu Natal and extends over 2,291.93 km^2^ (Gottschall et al., 2019; Davids et al., 2022). The climatic conditions are sub-tropical to tropical with higher elevations characterized by higher rainfall and lower mean temperature. This combination of climatic conditions has resulted in a wide range of indigenous plants (Cockburn et al., 2016). Osindisweni is a small rural community in the EMA, about 25 km north of Durban harbor, with 2,365 people in 396 recorded households (Capital, 2011). Maphephetheni Uplands is a larger rural community, about 30 km northwest of Durban harbor, with 16,000 people within 2,000 homesteads (Cockburn et al., 2016; Capital, 2011).

**Figure 1:**
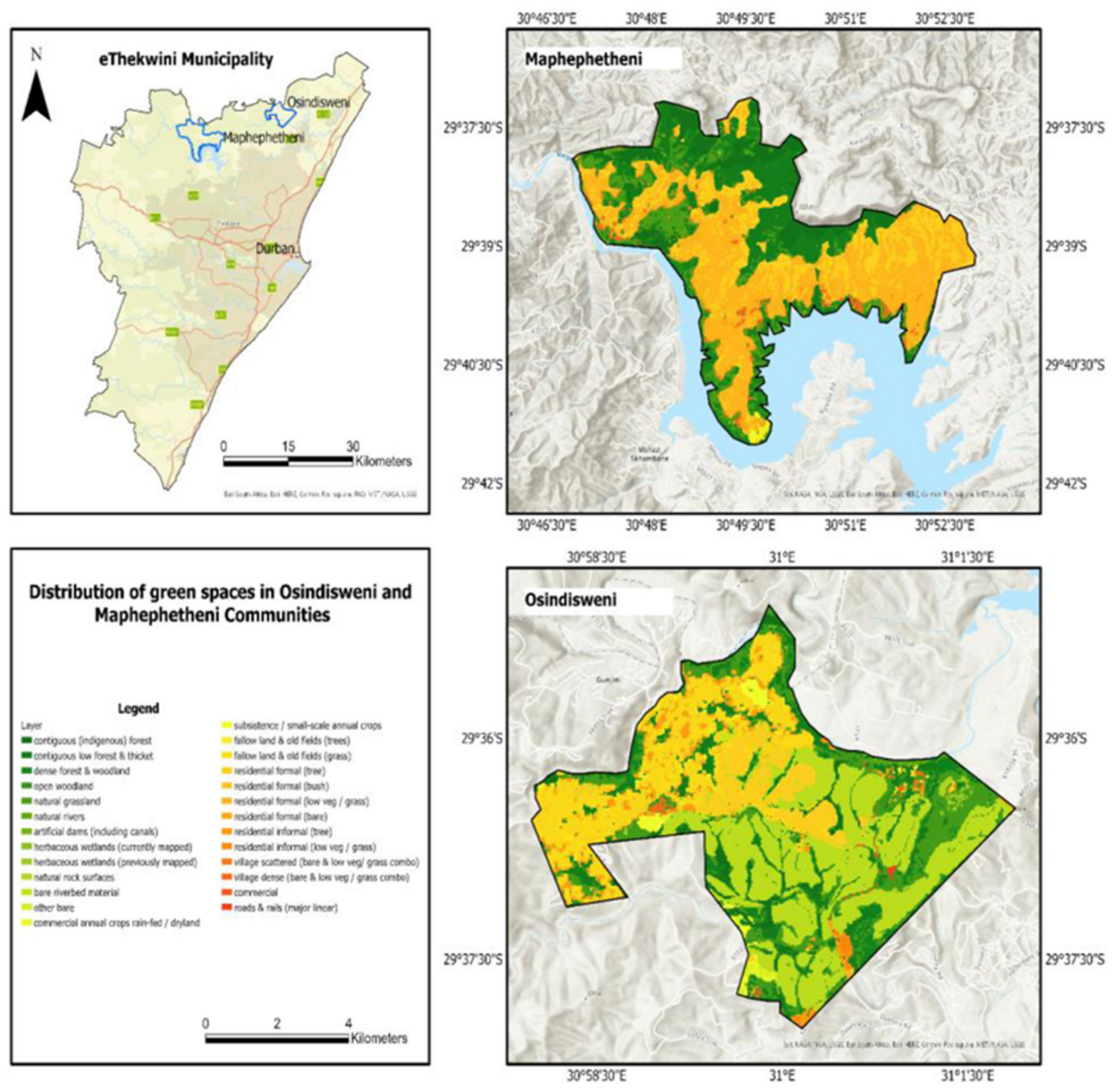
Map showing the distribution of green spaces within the Osindisweni and Maphephetheni study sites in KwaZulu-Natal, South Africa (Source: Author).

The two study sites were chosen because they are biologically diverse with multiple biomes (forests, grasslands, woodlands, and bushlands) (Shisanya and Hendriks, 2011; Scott-Shaw and Escott, 2011) (Figure 1). The locations are primarily inhabited by African people and are linked to extreme poverty, high unemployment, habitat degradation and food and nutrition insecurity (Munien et al., 2015).

### 2.2 Sampling

Osindisweni and Maphephetheni Uplands Communities were selected because of their poverty levels, food insecurity and the presence of local ecosystem projects, available to them. The Raosoft sample size calculation (2004) was used to calculate the sample size to provide for 90% confidence levels. The sample size for each study area calculated relative to the number of households, 75 households in Osindisweni and 205 households in Maphephetheni were randomly selected. In total, we managed to collect data from a total of 280 households from both communities.

### 2.3 Data collection

Between June and December 2021, questionnaires were used to collect quantitative data, while Focus Group Discussions (FGDs) and key informant interviews were conducted to collect qualitative data. Questionnaires included close-ended questions about demographics, the use of trees and green spaces, as well as on food and nutrition security, the FCS was also included in the questionnaire. Open-ended questions were asked during FGDs and key informant interviews to under understand in detail the use of trees and green spaces, and the perceptions of these. The survey respondents were either household head or acting household head. The questionnaires were administered by three bilingual (isiZulu and English) enumerators to ensure that respondents understand the questions, clearly. Ethical clearance for this study was obtained from the University of KwaZulu-Natal’s Human and Social Sciences Research Ethics Committee. All participants provided informed consent to participate in the study. Selected key informants included the ward councilors from both communities, an extension officer from Wildlands, a representative from the Wildlands with extensive knowledge of tress and green spaces knowledge, and traditional community leaders. One focus group was held in each community; on average, 10 people participated in the FGDs.

### 2.4 Data analysis

Statistical analyses were undertaken using the SPSS Version 27. Qualitative data from the focus groups and key informant interviews were analyzed by documenting and elucidating the participants’ meanings, experiences, and perspectives on the questions covered using the thematic analysis. Descriptive statistics was used to analyse household characteristics. The effect of trees and green spaces on the Food Consumption Score (FCS) (an index of household nutrition security) was then analyzed using the ordered logistic regression model.

#### 2.4.1 Food and nutrition security measurements (Food Consumption Score)

Food and nutrition security can be described and measured according to various definitions, dimensions, timeframes, and units of analysis. This study used the FCS to capture and classify food and nutrition information and guided appropriate responses. The FCS is a composite score that is determined by taking into account how many food groups (out of a possible eleven) a household consumed in the previous seven days (Akbay and Ahmadzai, 2020). The FCS is calculated through multiplying the number of days in a week that the food group was consumed against the weighting of that food group as determined by its nutritional significance (Fite et al., 2022). The calculations for this indicator only consider food consumed within the household.

Using a recall period of 7 days, the respondents were asked to indicate the frequency of consumption of different food groups. These food groups included: (i) cereals and grains; (ii) white roots and tubers; (iii) legumes, (iv) meat, (v) fish, (vi) milk and milk products, (vii) eggs; (viii) orange and dark yellow foods; (ix) dark green leafy vegetables and other vegetables, (x) fruits; and (xi) sugar, oil, and other condiments. Broad food groups and associated FCS weights were as follows: cereals and grains x2; White roots, tubers and legumes x3; Vegetables x1; Meat and fish x4; Milk x4; Sugar x0.5; Oil x0.5; Condiments were weighed at 0. Households were categorized into three categories using the FCS thresholds: Acceptable for satisfactory diets (> 35), Borderline for limited diets (Turner-Skoff and Cavender, 2019; Govender et al., 2017; Pradhan et al., 2018; Newton et al., 2020; Rasolofoson et al., 2018; Rasmussen et al., 2020; Baudron et al., 2019; Kehlenbeck and Jamnadass, 2014; Sardeshpande and Shackleton, 2020; Ellison et al., 2017; Gitz et al., 2021; Jabbar et al., 2021; Sivadas, 2022; Kushitor et al., 2022; Gottschall et al., 2019), and Poor for inadequate diets (<21) (Fite et al., 2022; Vaitla et al., 2015).

#### 2.4.2 The ordered logistic regression model

The determinants of FCS (household nutrition security) were analyzed using ordered logistic regression. In case of ordered logistic we introduce a latent variable *y*^∗^, which is not an observed variable; however, the properties of the variable are useful and intuitive (Chiphang and Singh, 2020).

*y* = 0; *if the household food consumption is poor*

*y* = 1; *if household food consumption is in the borderline*

*y* = 2; *if household food consumption is acceptable*

Thus, the latent continuous variable model specification (including the logistic error term) is described as;

$$y_{i}^{*}=\beta +\beta_{1}x_{1i}+\beta_{2}x_{2i}+\beta_{3}x_{3i}+\cdots \cdots \beta_{n}x_{ni}+\varepsilon_{i}$$

Whereas the observed ordered categorical variable *y*_*i*_ model specification is described as;

$$\begin{array}{c} \frac{pr\left(y_{i}>j\right)}{pr\left(y_{i}<j\right)}=\exp\{-\gamma_{j}+\beta_{o}+\beta_{1}x_{1i}+\beta_{2}x_{2i}+\beta_{3}x_{3i}\\ +\cdots \cdots \beta_{n}x_{ni}\} \end{array}$$

Where;

$$\begin{array}{r} y_{i}=\;food\;consumption\;\\ x_{ni}=\;determinants\;\\ \beta_{o}=\;intercept\;\\ \beta_{n}=\;coefficients\;to\;be\;estimated\;\\ \varepsilon_{i}=\;error\;terms\; \end{array}$$

The variables were inherently ordered by three different levels: poor diet, borderline diet and acceptable diet. By introducing the threshold variables of *y*_1_ and *y*_2_ we will be able to formulate the formal relationships between the latent $\left(y_{i}^{*}\right)$ and observed (*y*_*i*_) model specifications as;

$$\begin{array}{r} y_{i}=0\;if\;y_{i}^{*}\leq y_{1}\\ y_{i}=1\;if\;y_{1}\leq y_{i}^{*}\leq y_{2}\\ y_{i}=2\;if\;y_{i}^{*}>y_{2} \end{array}$$

Where *y* is an unobserved parameter that is estimated jointly with *β*.

The dependent variable was the FCS, where 0 was for poor, 1 for borderline, and 2 for acceptable. The food groups that had an effect on FCS, are given in Table 2. Table 1 below summarizes the variable names and definitions.

## 3 Results

### 3.1 Socio-demographic profiles of the respondents

The results show that more females than males participated in the study (Table 2). Most families were nuclear, 98.7% in Osindisweni and 94.1% in Maphephetheni. Approximately 65.8% and 57.4% of the households in Osindisweni and Maphephetheni were married, respectively. Regarding the level of education for both communities, most respondents attained secondary education. However, less than a quarter of the respondents had attained tertiary education in the form of a certificate or diploma. Less than 10% of the respondents indicated no formal education. Over 90% of the respondents had backyard gardens, however, the dominant source of regular means was markets in both communities.

### 3.2 Tree species harvested from green spaces

As shown in Table 2, above the respondents mentioned that they sourced and harvested food from trees and green spaces. A total of 30 species we commonly mentioned and below is a list (Table 3) showing tree species that were harvested from trees and green spaces.

### 3.3 Food groups consumed by households in the past seven days at Osindisweni and Maphephetheni communities

All households (100%) consumed cereals (Table 4). More than three-quarters (88.8%) of the respondents consumed legumes, which the FGDs confirmed were mainly dried beans. About two-thirds of the respondents indicated that they had consumed food from the following food groups: spices and condiments (76.1%), drinks (68.9%), sugar (73.6%), as well as oil and fats (72.5%). More than half of the respondents stated that they had consumed milk (60%), tinned fish (55%), eggs (60%), meat (54.3%), organ meat (55%), fruits including wild and indigenous fruits (61.1%), green vegetables including indigenous vegetables (58.2%) as well as other vegetables (66.1%).

### 3.4 Household food consumption situation

**Figure 2:**
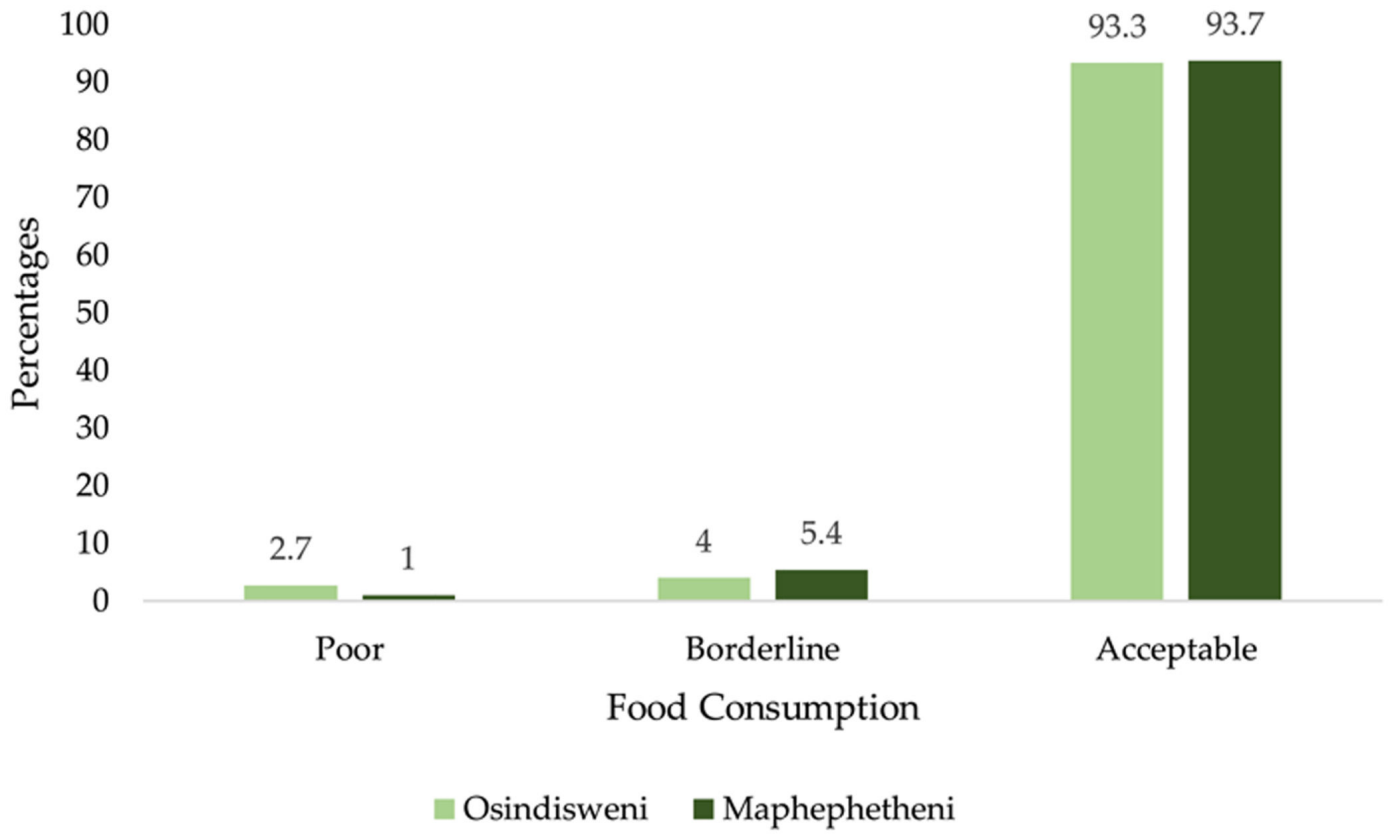
Household food consumption situation in Osindisweni and Maphephetheni areas, 2021.

The overall results of the FCS revealed that 93.6% of the households consumed “acceptable” diets, 5.0% were in the borderline while about 1.4% of the households consumed poor diets. The results from Osindisweni and Maphephetheni communities showed that almost all of the households (93.3% and 93.7%) had acceptable diets, while 4% and 5.4% were in borderline consumption, and 2.7% and 1.0% had poor diets, respectively (Figure 2).

### 3.5 Results of the ordered logistic regression

Both cultivated and uncultivated trees and green spaces, household size, number of dependants, number of dependents as well as access to training, agricultural assistance, extension, and advisory services significantly impacted household nutrition security (Table 5). Cultivated trees and green spaces negatively and significantly affected households’ nutrition security. Increasing food consumption from trees and green spaces will increase household nutrition security and would lead to a better diet.

### 3.6 Results from focus group discussions

A thematic analysis was conducted to uncover several key themes concerning household nutrition security. The analysis attempted to achieve an equal and fair representation of the opinions of the participants. Six main codes and 24 sub-codes were identified and merged into the three themes shown below. Representative quotes were selected to demonstrate study findings while retaining colloquial language. The analysis highlights the significance of low-cost food options such as beans in addressing household nutrition security. It also emphasizes the reliance on government grants and the need for additional assistance to ensure nutritious food access. Home gardens are seen as a potential solution, but challenges such as livestock damage must be addressed.

#### 3.6.1 Theme one: the affordability and consumption of beans

Participants in the focus groups indicated that beans are less expensive than meat and are more commonly consumed as a condiment with foods such as rice or pap. This suggests that beans play an important role in providing nutrition to households, particularly those experiencing food insecurity. One female community leader stated that,

*“We know the need for a balanced diet, but most families lack access to nutritious food and hence end up eating what is there to survive, which can be pap and beans for breakfast, lunch, and supper.”*

#### 3.6.2 Theme two: food and nutrition insecurity and reliance on government grants

Participants in the focus group expressed concern about food insecurity in their community. They stated that due to a lack of access to nutritious food, some people resort to scavenging food from dumpsites. One community leader mentioned,

*“We have a huge food problem in our community*∆*most families survive on grants from the government, and, usually, that money is not enough to sustain food needs; hence people suffer from hunger.”*

This clearly signifies the reliance on government grants for survival and the inadequacy of these grants in meeting households’ food needs.

#### 3.6.3 Theme three: the role of home gardens and challenges faced in gardening

The eThekwini municipality’s community liaison officer discussed the municipality’s efforts to address food and nutrition insecurity through the establishment of home gardens. They did, however, mention the difficulties they faced, particularly with livestock destroying crops if the garden is not fenced. This suggests that, while home gardens can help with household nutrition security, there are practical issues that must be addressed in order for them to be effective. According to the officer,

*“The only setback we have experienced is livestock destroying the crops planted, especially if the garden is not fenced.”*

## 4 Discussion

### 4.1 Contribution of trees and green spaces to household nutrition security

These results show that Osindisweni and Maphephetheni communities can be classified as having limited access to food, formal education, employment, and income-generating opportunities. While market purchases served as the primary source of regular meals, trees and green spaces also played a substantial role, particularly in Maphephetheni area (Govender et al., 2017). It is not surprising that trees and green spaces contribute substantially toward household nutrition since 88.4 % of the households had trees in their yards and produced food (vegetables, fruits, tubers (sweet potato and amadumbe-*Colocasia esculenta*), legumes and maize) in their home gardens and farms, which helped to diversify diets. In the same vein, the study of Tesfaye et al. (2008) found that households that grow and cultivate plants for food improved their food security status because of increased production, income, and consumption (Tesfaye et al., 2008). In South Africa, based on the various studies conducted in different regions of the country, cultivating plants for food through gardening and farming has increased livestock production, crop diversification and intensification. These outcomes, in turn, contribute to assured food security.

#### 4.1.1 Dietary diversity and nutrition

More than half of the respondents mentioned that they had eaten vegetables during the seven-day recall period. Most households had home gardens, which boosted the availability and accessibility of vegetables in the study area. Vegetable consumption can contribute to a higher-quality diet because vegetables are a good source of vitamins and minerals. Ochieng et al. (2017) support this and suggest that increasing nutritional diversity requires increased vegetable production. Less than half of the respondents indicated that they had consumed tubers in the form of sweet potatoes (44.3%) and orange-fleshed vegetables (20.7%) in the last seven days. This is consistent with the findings of Sibhatu et al. (2015) and Sambo et al. (2022); food groups such as fruits, milk and milk products, fish, meat and eggs were consumed by fewer respondents as compared to cereals. This could be due to low literacy levels and insufficient money to purchase nutritious foods. Households run by people without formal education lack knowledge about nutrition, the advantages of a healthy diet, and the funds to purchase nutritious foods. They are consequently less likely to consume a healthy diet than households with formal education who are probably employed and have funds to purchase nutritious foods. The FGDs also revealed that most people in the study area eat what is affordable, accessible and available from their gardens and farms. Furthermore, they revealed that nutrition is not a primary concern when buying or preparing food; they eat to survive. The most probable explanation for this food and nutrition insecurity is limited access to formal education, employment, and income-generating opportunities, which are all characteristics of the study areas.

#### 4.1.2 Food consumption patterns

Almost all the respondents were found to have acceptable diets while a very small % (≥5%) were in the borderline or consumed poor diets (Figure 2). These results are similar to those of other developing countries; when examining household food security in rural Zimbabwe, Butaumocho and Chitiyo (2017) found that just 8% of households had poor food consumption, 24% had borderline consumption, and 68% had acceptable consumption (Butaumocho and Chitiyo, 2017). Although a high prevalence of households had acceptable food consumption, these households largely consumed food from the food groups that do not fully meet nutritional needs such as cereals, drinks and sugars.

#### 4.1.3 Reliance on market purchases

The ordered logistic regression model revealed that five of the variables fitted into the model had a negative and statistically significant association with household food consumption, suggesting a decrease in nutrition security and poor diets. Both cultivated and non-cultivated green spaces had a significant negative relationship with household nutrition security. The results suggest that as cultivated and non-cultivated green spaces increase the likelihood of household nutrition security decreases with other variables in the held constant. This implies that the quality of diets and food consumption likely remains very low even when households include food from trees and green spaces in their diets. This result was surprising because foods from trees and green spaces are nutrient-dense and are expected to improve nutrition security and diets. However, even though households in the study area harvest and consume food from trees and green spaces, there was still a high reliance on purchased food. This could be as result of a lack of scientific knowledge on the available food resources found in forest trees and green space, use potential and their harvesting and preparation techniques (Akbay and Ahmadzai, 2020; Sambo et al., 2022).

#### 4.1.5 Household size and nutrition

The regression analysis also showed that household size and the number of dependents were significantly and negatively associated with household nutrition security. The implication of this results as depicted by the analysis shows that as households likely to decrease. The average number of people per household in this study was eight, with a total number of dependents ≥6 deemed large, while those with dependents <6 was considered average. This corresponds with Cheteni (2014) results which revealed that many economically inactive household members cause poor diets and nutrition. A large household size puts pressure on the availability and accessibility of food as there are more food and non-food expenses (Habte and Krawinkel, 2016).

#### 4.1.5 Access to support

Access to training, agricultural assistance and extension and advisory services were significantly negatively associated with household nutrition security, implying that access to extension services is likely to reduce the nutrition security status. The results show that as the probability of access to training, agricultural assistance and extension and advisory services increases, the chance of household nutrition security decreases with other variables in the held constant. This was not expected because agricultural training, assistance and extension is associated with the development and growth of smallholder farming and community gardens, ultimately leading to household nutrition security. Most households with access to training, assistance and extension services are expected to have improved access to diversified foods for consumption than households without access to training, assistance and extension (Ndlovu et al., 2022). Conversely, Sambo et al. (2022) found that extension and advisory services significantly impacted diets and nutrition security because extension services promote increased access to resources, leading to increased food production and income, which culminates in better purchasing power. This shows that access to extension services can improve food production and help households produce various foods for consumption and sale. However, despite the municipality’s interventions to implement community gardens, the findings indicate the need for targeted interventions to promote the consumption of trees and green spaces for more nutritious diets.

## 5 Conclusion and recommendations

Communities in the study area perceive trees and green spaces to be contributing toward nutrition security but these need to be supplemented. While the FCS of the respondents was characterized as largely acceptable, the diets of most households were still unbalanced, and most households consumed the cereals and legumes food groups. Uncultivated trees and green spaces, household size, number of dependants, as well as access to training, agricultural assistance, extension, and advisory services likely did not improve the nutrition security of the households. However, the potential to increase nutrient-dense diets in the study area justifies the need to work toward improving strategies that encourage accessing food from trees and green spaces. Collaborations between various stakeholders, including nutritionists, extensionists, and researchers, should be promoted to create a holistic approach to enhance household nutrition security through varied diets, including products from trees and green spaces, thus improving household diets.

## 6 Limitations

The study measured the household nutrition security through the use of FCS, further studies could be conducted to measure household nutrition security using both FCS and Household Dietary Diversity Score. More so, the assessment of household nutrition security using the FCS did not include a detailed list of food items sourced from trees and green spaces with those obtained from market purchases. The study was conducted in the KwaZulu Natal province, further studies could be carried out in the other provinces of South Africa to fully examine the contribution of trees and green spaces in the country.

## Figures and Tables

**Table 1:** Definitions and summary statistics of variables used in the ordered logistic regression model.

Variable name	Variable definition
Non-Cultivated green spaces	Any land covered by naturally occurring vegetation (i.e., grasslands, forests, parks etc.)
Cultivated green spaces	Any land covered by cultivated vegetation (i.e., croplands and gardens)
Local trees	Naturally occurring trees that add value to households in their vicinity
Wealth index: non-agricultural related assets	Measure of wealth in the form of non-agricultural assets (i.e., furniture and vehicles)
Wealth index: agricultural-related assets	Measure of wealth in the form of agricultural assets (i.e., equipment)
Gender of household head	Gender of household head (male/female)
Age of household head	Age of household head (respondent) in years
Marital status of household head	Marital status of household head
Household size	Number of people living in that particular household
Education level of the head of household	Level of education of household head (primary/secondary/tertiary)
Number of dependents	Number of children depending on household head for their livelihood
Monthly income	Money received by household head monthly
Grants	Money received from social grants
Access to training, agricultural assistance, extension, and advisory services	Access to any form of agricultural training or advisory services

**Table 2:** Socio-demographic profiles of the sampled households, 2021.

	Osindisweni	Maphephetheni
**Sex of the respondents**		
Male	18.4%	26.5%
Female	81.6%	73.5%
**Household composition**		
Nuclear	98.7%	94.1%
Polygamous	1.3%	5.9%
**Level of education**		
Primary	14.5%	20.6%
Secondary	63.2%	56.6%
Tertiary	13.2%	13.2%
No education	9.2%	9.3%
**Marital status**		
Married	65.8%	57.4%
Single	18.4%	23%
Separated	6.5%	7.4%
Widowed	9.2%	12.3%
**Source of regular meals**		
Non-cultivated green spaces (forests, woodlands, bushlands, grasslands, parks)	12%	28.8%
Cultivated green spaces	2%	2.9%
Communal gardens	90.7%	93%
**Backyard gardens**		
Bought from markets	64%	51.7%
From friends and relatives	24%	19.5%

Own analysis.

**Table 3:** List of tree species harvested by respondents from green spaces seasonally.

Number	Species Latin name	Species English name	Species local name	Uses	Where it’s grown or harvested	Season
1	*Annona senegalensis*	Wild Custard Apple	Umphofu	Wood, medicine, etc	Non-cultivated green spaces	June
2	*Berchemia discolor*	Birdplum, Brown ivory	uMadlozane, uMhlungulo, uVuku	Medicine	Gardens	Jan-Jul
3	*Berchemia zeyheri*	Red ivory wood, Purple ivory, Pink ivory	umNcaka, umNini	Wood, medicine, etc	Non-cultivated green spaces	Jan-Apr
4	*Carissa bispinosa and edulis*	Num num	aMantungulu	Medicine	Gardens	Mar-Oct
5	*Carissa macrocarpa*	Natal plum	Amatungulu	Food, medicine, etc	Gardens	Mar-Oct
6	*Cucumis africanus and metuliferus*	Wild cucumber, Wild gherkin, Jelly melon	iSende-lenja, uSelwa-lwemamba	Medicine	Non-cultivated green spaces	Feb-Jul
7	*Dovyalis caffra*	Kei-apple	uMqokolo	Fodder	Gardens	January
8	*Eriobotrya japonica*	Japanese medlar; Japanese plum	Amanumbela	Food	Gardens	Sep-Jan
9	*Ficus craterostoma and sur*	Forest fig, Forest strangler-fig, Blunt-leaved forest fig, Bastard Natal fig, Broom cluster fig	uMthombe, uMbombe, iSihlamfane, uMkhiwane	Food, medicine, etc	Non-cultivated green spaces	Jul-Dec
10	*Garcinia livingstonei*	African mangosteen	umPhimbi, uGobandlovu	Medicine	Non-cultivated green spaces	
11	*Harpephyllum caffrum*	Wild plum	umgwenya	Wood, medicine, etc	Gardens	Mar-Jun
12	*Hyphaene coriacea*	Lala palm, Gingerbread tree, Fan palm	iLala	Food, medicine, etc	Non-cultivated green spaces	All year
13	*Landolphia* *(Ancylobotris) capensis*	Rock milk Apricot, Wild apricot, Dwarf wild apricot, Wild peach	Mdongwe	Food	Non-cultivated green spaces	Dec-Feb
14	*Mimusops zeyheri*	Transvaal red milkwood	uMpushane	Food	Gardens	Apr-Sep
15	*Opuntia engelmannii andficus-indica*	Prickly pear	uMthelekisi	Food	Gardens	Nov-Feb
16	*Osyris compressa*	Cape sumach, coastal tannin bush	uMbulanyathi	Food	Non-cultivated green spaces	Apr-Dec
17	*Passiflora edulis*	Passion fruit, granadilla	iJembuluka	Food	Gardens	Oct-Jan
18	*Phoenix reclinate*	Wild date palm	iSundu	Fiber	Non-cultivated green spaces	Feb-Apr
19	*Psidium cattleianum and guajava*	Strawberry guava; Guava	uGwava	Food	Gardens	All year
20	*Sclerocarya birrea*	Marula	uMganu	Food, medicine, etc	Gardens	Feb-Jul
21	*Strychnos madagascarensis and spinosa*	Black monkey orange; Green/spiny monley orange	umGluguza, umKwakwa	Wood, medicine, etc	Non-cultivated green spaces	All year
22	*Syzygium cumini and jambos*	Jambolan, Rose apple	uMdoni	Food	Gardens	All year
23	*Tabernaemontana* *elegans*	Toad tree	uMKhahlwana, umKhadlu	Food	Non-cultivated green spaces	Mar-Jun
24	*Vangueria infausta*	Wild medlar	umViyo, umTulwa	Medicine	Non-cultivated green spaces	Jan-Apr
25	*Ximenia caffra and americana*	Large sourplum	umThunduluka-obmvu	Medicine	Non-cultivated green spaces	Nov-Feb
26	*Moras alba*	Mulberry	uMabhulosi	Fodder	Gardens	All year
27	*Amarantus cruentus*	Amaranth	Imbuya, Imfino	Food	Gardens	All year
28	*Biden Pilosa*	Blackjack	uMhlabangubo, ilenjana	Food	Non-cultivated green spaces	All year
29	*Colocasia esculenta*	Taro	Amadumbe	Food	Gardens	All year
30	*Trichilia emetica*	Natal mahogany	uMukuhlu	Wood, medicine, etc	Non-cultivated green spaces	Jan-May

**Table 4:** Food groups consumed by households in the Osindisweni and Maphephetheni communities in the preceding seven days.

Number	Food Group	Percentage Of respondents
01	Cereals: maize, rice, wheat, sorghum, millet, and any other foods made from cereals such as porridge, bread, and noodles	100
02	White roots and tubers: Potatoes, white sweet potato, and cassava	44.3
03	Orange-flesh vegetables: Pumpkin, carrot, butternut, or sweet potato	20.7
04	Dark green leafy vegetables, including wild/indigenous vegetables	58.2
05	Other vegetables (tomato, onion, green beans, gem squash, eggplant, including wild/indigenous vegetables)	66.1
06	Orange-colored fruit (e.g., ripe mango, apricot, spanspek, papaya, dried peach and 100% fruit juice made from these)	64.3
07	Other fruit (e.g., oranges, banana, apple, pear etc.), including wild/indigenous fruits	61.1
08	Organ meat (liver, kidney, heart or other organ meats or blood-based foods)	55
09	Meat (e.g., beef, goat, sheep, poultry, pork, fish, insects)	54.3
10	Eggs from any animal	60
11	Fish and seafood (fresh, tinned, or dried and shellfish)	55
12	Legumes, nuts, and seeds (dried beans, dried peas, lentils, nuts, peanuts, seeds) or foods made from these (e.g., peanut butter)	88.8
13	Milk and milk products (Milk, sour milk, cheese, yogurt, custard, or any other milk products, or any drinks made with milk)	60
14	Oils and fats (e.g., sunflower, rama, lard, butter added to food or used for cooking)	72.5
15	Sugars and sweets (e.g., sugar, sweets, chocolates, cake and sweetened biscuits, honey, jam, sugar sweetened drinks e.g., cold drinks, sugary foods, sweetened condensed milk)	73.6
16	Spices and condiments (e.g., spices, salt, pepper, etc.), condiments (e.g., chutney, tomato sauce)	68.9
17	Drinks (Coffee, tea, cocoa)	76.1

Own analysis.

**Table 5:** Determinants of the FCS using the ordered logistic regression.

FCS	Coef.	St. Err.	*t*-value	*p*-value
Non-Cultivated green spaces	−0.377	0.229	−1.65	0.099*
Cultivated green spaces	−0.516	0.270	1.91	0.056*
Local trees	0.158	0.229	0.69	0.490
Wealth Index: non-Agricultural Related Assets	0.311	0.304	1.02	0.307
Wealth Index: agricultural-Related Assets	0.378	0.237	1.60	0.110
Gender of household head	−0.751	0.823	−0.91	0.361
Age of household head	0.014	0.022	0.63	0.529
Marital status of household head	−0.307	0.196	−1.57	0.117
Household size	−0.223	0.122	−1.82	0.068*
Education level of the head of household	0.104	0.349	0.30	0.767
Number of dependents	−0.297	0.160	1.85	0.064*
Monthly income	0.000	0.000	−0.52	0.604
Grants	−1.022	1.011	−1.01	0.312
Access to training, agricultural assistance, extension, and advisory services	−2.091	0.863	−2.42	0.015*
Cut1	−7.770	2.107	.b	.b
Cut2	−5.435	1.964	.b	.b
Mean dependent var	2.936			
Pseudo r-squared	0.167			
Chi-square	22.457			
Akaike crit. (AIC)	144.258			
Bayesian crit. (BIC)	202.415			
Prob > chi^2^	**0.070**			

Results of the ordered logistic regression (Source: Own analysis). ^∗^p < 0.1.

## Data Availability

The raw data supporting the conclusions of this article will be made available by the authors, without undue reservation.
